# Application of whole genome sequencing for carrier and diagnostic assessment of spinal muscular atrophy in Taiwan

**DOI:** 10.1038/s41525-025-00524-1

**Published:** 2025-10-29

**Authors:** Li-Ling Lin, Pei-Miao Chien, Tzu-Hung Hsiao, Han-Yu Ye, Shu-Hsuan Liu, Tsang-Ming Ko, Chien-Hao Huang, Pei-Lung Chen, Wen-Chun Chen, Yuh-Tsyr Chou, Chao-Szu Wu, Hung-Hsin Chen, Yin-Hsiu Chien, Jacob Shu-Jui Hsu

**Affiliations:** 1 Department of Obstetrics and Gynaecology, Taichung Veterans General Hospital, Taichung, Taiwan; 2 Graduate Institute of Molecular Medicine, College of Medicine, National Taiwan University, Taipei, Taiwan; 3 Graduate Institute of Medical Genomics and Proteomics, College of Medicine, National Taiwan University, Taipei, Taiwan; 4 Department of Medical Research, Taichung Veterans General Hospital, Taichung, Taiwan; 5 Genephile Bioscience Laboratory, Ko’s Obstetrics and Gynaecology, Taipei, Taiwan; 6 Department of Medical Genetics, National Taiwan University Hospital, Taipei, Taiwan; 7 Institute of Biomedical Sciences, Academia Sinica, Taipei, Taiwan; 8 Department of Paediatrics, National Taiwan University Hospital and National Taiwan University College of Medicine, Taipei, Taiwan

**Keywords:** Genome informatics, High-throughput screening, Sequence annotation, Population genetics, Sequencing, Preventive medicine, Neurodegeneration, Genetic counselling

## Abstract

This study aimed to evaluate the feasibility of whole-genome sequencing (WGS) combined with computational tools for spinal muscular atrophy (SMA) carrier screening and disease diagnosis in Taiwan. WGS data from 1492 Taiwan Biobank participants and two patients with SMA were analysed to determine the *SMN1* and *SMN2* copy numbers using Illumina DRAGEN SMN Caller and validated by multiplex ligation-dependent probe amplification (MLPA). Among 1480 samples analysed, 23 SMA carriers were identified, yielding a carrier frequency of 1.55%. MLPA confirmed the accuracy of *SMN1* and *SMN2* copy number results detected using WGS. Both patients with SMA presented compound heterozygous variants with one *SMN1* copy loss and the other *SMN1* variant, specifically *SMN1*,c.815A>G, and *SMN1*,c.81+2_81+3delTG, respectively. Taken together, combining WGS with advanced bioinformatics tools is a feasible and promising approach for SMA carrier screening and disease diagnosis.

## Introduction

Spinal muscular atrophy (SMA) is a severe neuromuscular disorder caused by the degeneration of spinal motor neurons, presenting with muscle atrophy and progressive muscle weakness. SMA follows an autosomal recessive mode of inheritance, primarily due to homozygous deletions in the *SMN1* gene located on chromosome 5q13.2. *SMN1* and *SMN2* share high sequence similarity but differ by several nucleotides, including a critical change in exon 7 that affects splicing. While both genes are functional, pathogenic variants in *SMN1* have a greater impact because *SMN2* predominantly produces truncated, less effective SMN protein. Approximately 5% of cases result from non-deletion variants or compound heterozygosity of *SMN1*^1^. It is one of the most common genetic causes of neonatal death, with an incidence of 1 in 5000 to 10,000 live births^2,3^. Even though a recent clinical trial demonstrated the potential for prenatal treatment of fetuses with SMA^4^, carrier screening remains a crucial approach to preventing this devastating disease.

In East Asia, SMA carrier frequency ranges from 1.24% to 3.97% (Table S1). Carrier screening is essential for identifying at-risk couples and enabling informed reproductive decisions. In Taiwan, the largest study of pregnant women reported a carrier frequency of 2.10%^2^. Current screening relies on multiplex ligation-dependent probe amplification (MLPA)^1,5^, which is highly sensitive and specific for detecting *SMN1* deletions. However, this approach has limitations in detecting single-nucleotide variants (SNVs) or complex variants, which can result in false-negative carrier screening results.

Next-generation sequencing (NGS) and whole-genome sequencing (WGS) have revolutionised genetic screening by enabling simultaneous analysis of multiple genes. However, the high sequence similarity between the *SMN1* and *SMN2* genes poses significant challenges in detecting pathogenic variants and understanding the structural complexities within these homologous regions^6,7^. Recent studies have demonstrated the potential of sequencing-based methods to improve SMA carrier detection rates. These include identifying silent carriers with the “2 + 0” genotype, where an individual has two *SMN1* copies on the same chromosome and specific loss-of-function variations^8–12^.

This study aimed to evaluate the feasibility of WGS combined with computational tools for SMA carrier screening and disease diagnosis in Taiwan. Using data from the Taiwan Biobank (TWB)^13^, we analysed the carrier status for SMA-related variants and compared the performance of sequencing-based approaches with MLPA. Our findings will provide valuable insights into integrating advanced genomic technologies into routine carrier screening programs, paving the way for more comprehensive and equitable genetic testing.

## Results

### Demographic analysis

The demographic characteristics of the participants with WGS data from the TWB are summarised in Table 1, which also presents the demographic distribution of SMA carriers and non-carriers. The 1492 participants exhibited a uniform distribution of sex and age. Notably, most participants reported paternal and maternal ancestry of Holo descent, at 62.33% and 70.98%, respectively.

**Table 1:** Demographic characteristics of the 1492 WGS data in this study

	Overall numbers *n* = 1492	SMA carriers *n* = 23	Non-carriers *n* = 1457	*P* -value
Sex				
Male	744 (49.87)	10 (43.48)	732 (50.24)	
Female	748 (50.13)	13 (56.52)	725 (49.76)	0.665
Age (years)				
30−39	366 (24.53)	7 (30.43)	357 (24.50)	
40−49	359 (24.06)	4 (17.39)	352 (24.16)	
50−59	425 (28.49)	2 (8.70)	419 (28.76)	
≥60	342 (22.92)	10 (43.48)	329 (22.58)	0.041
Ancestry of participants				
Holo	876 (59.07)	15 (65.22)	856 (59.12)	
Hakka	105 (7.08)	1 (4.35)	102 (7.04)	
Mainlander	245 (16.52)	4 (17.39)	239 (16.51)	
Holo/Hakka	62 (4.18)	1 (4.35)	60 (4.14)	
Holo/Mainlander	164 (11.06)	2 (8.70)	160 (11.05)	
Hakka/Mainlander	29 (1.96)	0	29 (2.00)	
Holo/Hakka/Mainlander	2 (0.13)	0	2 (0.14)	1.000

The sex and age of these 1492 WGS data are evenly distributed. The main difference is that the participants’ paternal or maternal ancestry predominantly traces back to Holo. The distribution of carriers and non-carriers shows a statistically significant difference only in age, possibly due to an uneven distribution of the samples.

WGS whole-genome sequencing, SMA spinal muscular atrophy.

### *SMN1/SMN2* copy number calls for TWB WGS data

**Fig. 1:**
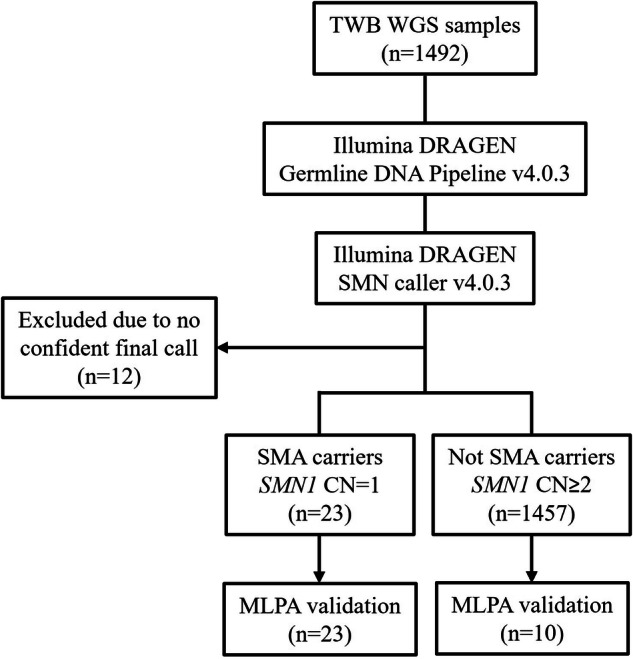
Analysis workflow for SMA carrier identification. Among the 1492 WGS data, 12 were excluded due to the output displaying “None”. We identified 23 SMA carriers, resulting in a carrier rate of 1.55%. A subset of participants with SMN Caller results was further validated using MLPA. WGS whole-genome sequencing, SMA spinal muscular atrophy, SMN survival motor neuron, CN copy number, MLPA multiplex ligation-dependent probe amplification.

Our SMA carrier analysis workflow and the corresponding results are illustrated in Fig. 1. Out of 1492 WGS data, 12 were excluded due to outputs showing “None,” indicating that the *SMN1* and *SMN2* copy number could not be determined. This occurs when there is insufficient or ambiguous read evidence at key paralog-specific positions, possibly due to low coverage or mapping quality in the *SMN* region. Among the remaining 1480 WGS data, we detected 23 SMA carriers with only one *SMN1* copy number. Homozygous *SMN1* deletion was not detected. The carrier rate was 1.55% (23/1480), meaning 1 in every 64 individuals in this study was a carrier. There was no statistical significance of carrier frequency between different sexes and among different ancestry groups.

The detailed distribution and frequency of *SMN1* and *SMN2* copy numbers are presented in Table 2. 91.82% (1359/1480) of the TWB participants had two copies of the *SMN1* gene, 6.42% (95/1480) had three copies, and only 0.20% (3/1480) had four copies. The most frequent *SMN1*:*SMN2* copy number types were 2:2 (55.27%) and 2:1 (30.81%). The copy number types of heterozygous SMA carriers included 1:2, 1:3, and 1:4. Only one *SMN2*Δ7-8 variant carrier was found. This aligns with previous literature^14^, indicating that this variant is less common in the Asian population.

**Table 2:** Distribution of *SMN1* and *SMN2* copy number types in our study

*N* (%)	*SMN1* CN0	CN1	CN2	CN3	CN4	Total
*SMN2* CN0	0	0	51 (3.45)	9 (0.61)	1 (0.07)	61 (4.12)
CN1	0	2 (0.14)	456 (30.81)	53 (3.58)	1 (0.07)	512 (34.59)
CN2	0	9 (0.61)	818 (55.27)	27 (1.82)	1 (0.07)	855 (57.77)
CN3	0	12 (0.81)	34 (2.30)	6 (0.41)	0	52 (3.51)
CN4	0	0	0	0	0	0
Total	0	23 (1.55)	1359 (91.82)	95 (6.42)	3 (0.20)	1480

Twelve samples were excluded due to their outputs showing “None”. The most common *SMN1*:*SMN2* copy number types were 2:2 and 2:1. Heterozygous SMA carriers exhibited copy number types of 1:2, 1:3, and 1:4. No cases of homozygous *SMN1* deletion were detected.

*SMN* survival motor neuron, *CN* copy number.

### Validation of the results from the SMN Caller

MLPA was used to validate the *SMN1* and *SMN2* copy number results derived from WGS for 33 samples, comprising all 23 identified SMA carriers and 10 selected non-carrier samples. The results were 100% concordant between WGS and MLPA. However, MLPA identified gene conversion events in *SMN2* exon 8 in two samples, which were not detected by the WGS-based method. In both cases, the exon 8 copy number was higher than that of exon 7, likely due to sequence conversion, which caused exon 8 to be misinterpreted as *SMN1*-like by MLPA probes (Fig. S1, 1). As MLPA relies primarily on exon 7 for interpretation, the final result was unaffected. The SMN Caller could not detect this discrepancy because its main differentiating sites did not include exon 8. Integrative Genomics Viewer (IGV) review (Figs. S1, 2) further supported this explanation, showing increased read depth near *SMN1*-specific positions in exon 8 compared to exon 7.

### *SMN1/SMN2* copy number analysis for patients with SMA

**Fig. 2:**
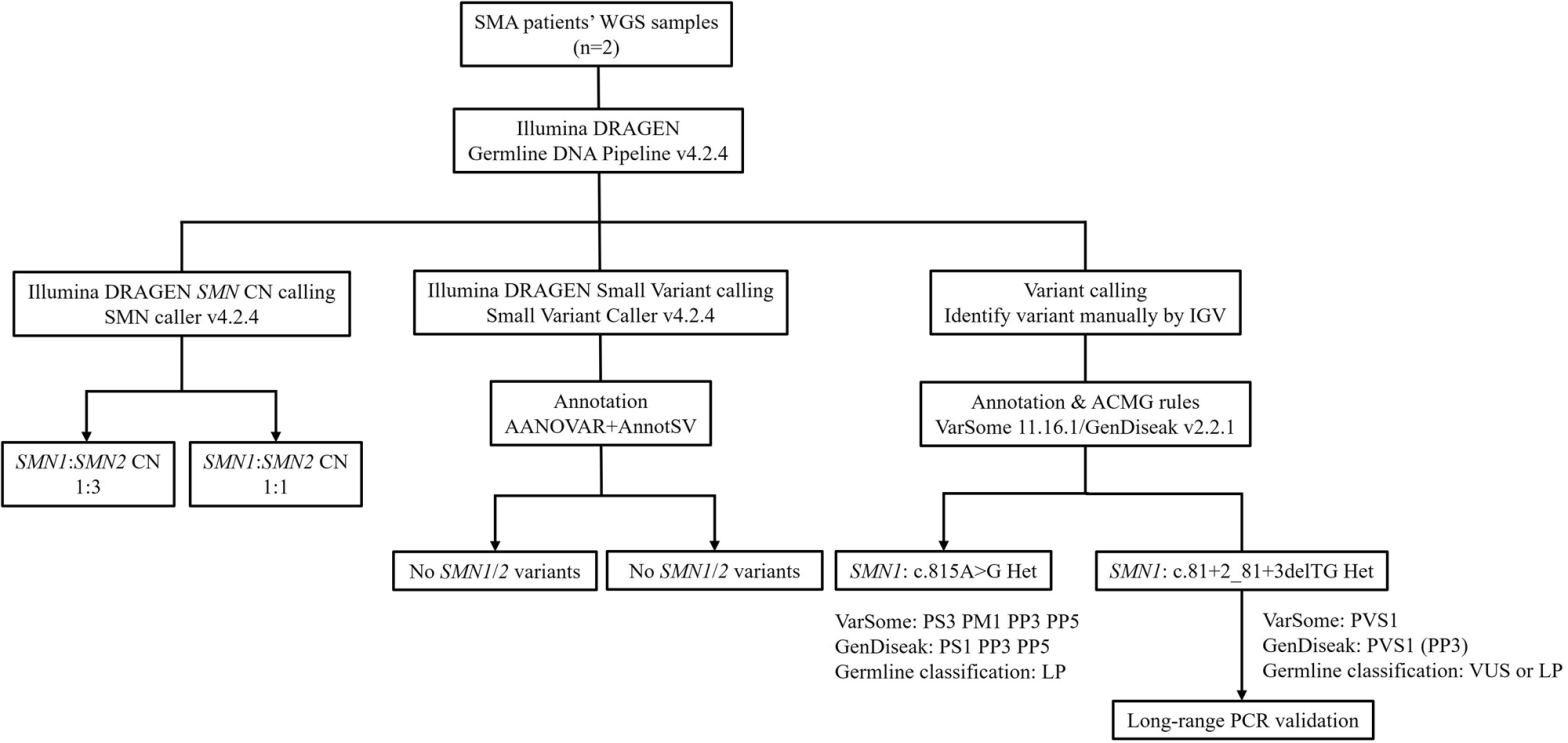
Analysis workflow for clinical patients with SMA. WGS data from two patients with SMA were analysed using the Illumina DRAGEN SMN Caller and Germline Variant Callers, followed by examination with IGV. Both patients were identified as compound heterozygous, carrying a single *SMN1* deletion on one allele and a small variant on the other *SMN1* allele. WGS whole-genome sequencing, SMA spinal muscular atrophy, SMN survival motor neuron, CN copy number, IGV Integrative Genomics Viewer, LP likely pathogenic, VUS variant of uncertain significance; PCR, polymerase chain reaction.

The SMA patient analysis workflow and results are presented in Fig. 2. The DRAGEN SMN Caller detected a single *SMN1* copy loss in both patients, indicating carrier status; however, the DRAGEN Small Variant Caller failed to identify additional variants. Further manual review using IGV revealed 16 SNVs and six insertions/deletions (indels), including one synonymous variant, 19 intronic variants, one missense variant, and one splicing variant. Among these variants, seven had a minor allele frequency (MAF) < 0.05, and five had an MAF < 0.01.

Two potentially pathogenic variants were identified in each patient: *SMN1* c.815A>G and *SMN1* c.81+2_81+3delTG. The *SMN1* c.815A>G variant, annotated as a missense variant, was observed at a variant allele frequency of 31% in this individual’s sequencing reads and is considered likely pathogenic (Fig. S2). This classification is supported by records from the ClinVar database, functional predictions, and bioinformatics analyses indicating a deleterious effect (MetaRNN score: 0.9985; Meta score: 20). Moreover, the variant occurs at a highly conserved genomic position, as evidenced by a PhyloP100Way conservation score of 8.495 and a GERP + +Rs score of 3.43, suggesting evolutionary constraint, and is rare in the general population, further reinforcing its potential pathogenicity.

Conversely, the *SMN1* c.81+2_81+3delTG variant (Fig. S3) remains of uncertain significance due to conflicting classifications: VarSome^15^ annotates it as likely pathogenic, while GenDiseak classifies it as a variant of uncertain significance (VUS). Both GenDiseak and VarSome viewed the variant as very strong evidence of being pathogenic, considering that this splice donor site variant may disrupt normal splicing and cause a loss of function. SpliceAI analysis also yielded a DS_DG score of 0.19 and a DS_DL score of 0.99, suggesting a high likelihood of donor site loss. However, GenDiseak strictly adheres to American College of Medical Genetics and Genomics (ACMG) guidelines^16^ and classifies the variant as VUS due to insufficient supporting evidence, whereas VarSome utilises a point-based Bayesian system and concludes it as likely pathogenic if no adjustment is made based on other evidence.

In summary, both cases were identified as compound heterozygote patients, carrying an *SMN1* copy number loss on one allele and an *SMN1* pathogenic SNV or indel on the other allele.

## Discussion

This study represents the first population-level analysis of SMA carrier frequency in Taiwan using WGS. We analysed *SMN1* and *SMN2* copy numbers in 1480 individuals from the TWB and identified 23 SMA carriers, yielding a carrier frequency of 1.55%. This rate is consistent with the reported carrier frequency range of 1.24–3.97% among East Asian populations (Table S1), suggesting genetic consistency across the region. However, it is slightly lower than the 2.1% carrier rate reported in a previous Taiwanese study based on a larger sample size (Table S3). A two-sample Kolmogorov–Smirnov test comparing copy number distributions showed no statistically significant difference (D = 0.36, *p* = 0.05863), indicating that the observed discrepancy may result from sampling variation rather than true population-level differences.

Despite the high accuracy of the Illumina DRAGEN SMN Caller in copy number detection—as confirmed by 100% concordance with MLPA in our validation set—variant calling in paralogous regions such as *SMN1* and *SMN*2 remains a major technical challenge. Due to high sequence homology and low mapping quality, the DRAGEN Germline Caller failed to detect small variants across the full *SMN* gene regions. To address this, we employed IGV and applied VarScan (v2.4.6)^17,18^ with multi-sample calling to enhance sensitivity. Using default filtering parameters—including minimum coverage of 10, at least two supporting reads, minimum MAF of 0.2, an average base quality of 20, and a p-value threshold of 0.1—this approach successfully identified several SNVs and indels in *SMN1* and *SMN2*, including pathogenic variants. Furthermore, while not applied in this study, tools such as Chameleolyser^19^ also offer promising solutions for paralogous region analysis by masking pseudogenes during alignment to improve mapping accuracy.

We identified compound heterozygous variants in two patients with SMA. Each patient exhibited a single *SMN1* copy loss in combination with a pathogenic SNV or indel on the remaining allele: *SMN1*:c.815 A > G and *SMN1*:c.81+2_81+3delTG, respectively. The pathogenicity classification of the latter variant remains conflicting. Both GenDiseak and VarSome recognized their strong loss-of-function potential (PVS1). However, GenDiseak, which strictly follows ACMG guidelines, classified the variant as VUS because a single PVS1 criterion is insufficient for likely pathogenic classification without additional evidence. Although the variant’s SpliceAI score (DS_DL = 0.99) supports splicing disruption (PP3), GenDiseak excludes PP3 when PVS1 is already applied, in line with ClinGen recommendations. In contrast, VarSome’s point-based Bayesian framework^20^ awarded sufficient points for a likely pathogenic label. This discrepancy underscores the complexity of variant interpretation and the limitations of guideline rigidity when applied to complex loci.

Several limitations should be noted. First, only 10 non-carrier samples underwent MLPA confirmation, leaving most of the cohort unvalidated. Second, although clinically relevant variants were detected in the two SMA patients, the cohort lacked cases with homozygous *SMN1* deletions—the most common SMA genotype—thus limiting diagnostic generalisability. Third, high sequence similarity between paralogous regions continues to complicate variant calling, and optimal parameters for accurate detection in these regions remain undetermined. Notably, MLPA detected two gene conversion events involving *SMN2* exon 8 that were missed by WGS. This reflects a limitation of the DRAGEN SMN Caller, which uses eight differentiating sites that do not include exon 8 (Supplementary Fig. S1, 2). Another limitation lies in the reliance on copy number for carrier classification. This approach cannot detect “2+0” silent carriers, who represent a significant source of false negatives in both MLPA- and WGS-based screening. As our cohort lacked phasing data, parental samples, and clinical follow-up, we were unable to assess this subset, and genotype–phenotype correlations were limited to the information from the questionnaire.. Additionally, pathogenic SNVs and structural variants beyond *SMN1* copy loss were not systematically screened, possibly underestimating the true carrier rate. Long-read sequencing technologies may help resolve current detection gaps and should be explored in future studies. Therefore, more comprehensive approaches combining WGS, long-read sequencing, haplotype phasing, and functional validation are needed to improve carrier screening performance and diagnostic accuracy. One further limitation is that the TWB is not specifically designed to collect couple-based cohorts. Our study is not a real-world implementation of reproductive screening but rather a methodological evaluation using TWB population-based data. Future studies using preconception or reproductive cohorts will be needed to address this gap.

Although our study focused on technical validation, future implementation of WGS-based SMA carrier screening must also consider cost and scalability. Recent literature suggests that WGS offers increasing diagnostic and economic advantages, particularly when integrated into multi-gene carrier panels. Compared to genotyping arrays, WGS demonstrates up to 2.4-fold higher sensitivity in identifying at-risk couples and may offer cost savings^21^. Meta-analyses have also shown WGS to be more cost-effective than WES or chromosomal microarray in diagnostic settings^22^. Broader carrier screening models using WGS may reduce lifetime costs per affected birth, though formal economic evaluations for general population screening remain limited^23,24^. As sequencing costs decline, further studies are needed to evaluate long-term outcomes, cost-utility, and healthcare system impact. While a full cost-effectiveness analysis is beyond the scope of this study, we highlight this as a critical area for future research.

Overall, this study demonstrates the feasibility of using WGS for SMA carrier screening in a real-world population setting. Integrating bioinformatics tools enabled accurate detection of *SMN1* and *SMN2* copy numbers and identified pathogenic variants in clinically confirmed SMA cases. These findings provide a framework for future development of robust, scalable genetic screening programs. As sequencing technologies continue to evolve, refining variant detection and interpretation methods will be key to advancing population genomics and precision medicine for rare genetic diseases like SMA.

## Methods

This study was conducted in accordance with all relevant ethical regulations and the principles of the Declaration of Helsinki. Ethical approval was obtained from the Biomedical Science Research Institutional Review Board of Academia Sinica, Taiwan (IRB-BM), and the Ethics and Governance Council of Taiwan Biobank (reference number ASIRB01-18041(N), July 25, 2022). All raw sequencing data were generated as part of the Taiwan Biobank project. No individual participant’s information was disclosed, and none of the results can be used to identify individual participants.

### Study materials

The Taiwan Biobank (TWB) is a nationwide, government-supported prospective cohort study designed to collect phenotypic and genomic data on the general Taiwanese population. Since 2012, TWB has enroled over 200,000 cancer-free adults aged 20 to 70 years from more than 30 recruitment sites across Taiwan, stratified by population density and region. This cohort accounts for approximately 0.87% of Taiwan’s total population and is broadly representative in demographic composition. While not explicitly designed for carrier screening, the TWB cohort provides a valuable, population-representative resource for evaluating genome-based screening approaches for autosomal recessive conditions.

For this study, around 2000 participants were randomly selected for WGS, ensuring a matched distribution of city of origin, self-identified ethnicity, age, and sex. From this subset, 1492 samples with complete WGS and metadata were included in our analysis. DNA extracted from blood samples was sequenced on Illumina HiSeq 2500, 4000, and NovaSeq 6000 platforms, generating approximately 90 GB of data per sample with an average coverage of 30x^25^. Additionally, WGS data from two clinical patients with SMA were obtained and sequenced on NovaSeq 6000, producing about 80 GB per sample with an average coverage of 45x and at least 94% of the genome covered at 20x or higher. Written informed consent was obtained from all participants, including those enroled in the TWB and the two clinical patients with SMA. This study was approved by the Academia Sinica Institutional Review Board (ASIRB01-18041(N)).

### Variant calling, annotation, and validation for SMA carriers and patients

We analysed the TWB’s WGS data and those of the two patients with SMA using Illumina’s DRAGEN DNA pipeline (versions 4.0.3 and 4.2.4, respectively). The reads of each sample in FASTQ format were aligned to the human reference genome (GRCh38, hs38DH) using the DRAGEN Bio-IT Platform’s ultra-rapid mapper, which employs the Smith-Waterman algorithm for precise alignment. The alignment files were sorted by genomic coordinates, and duplicate reads were marked using DRAGEN’s duplicate marking feature.

*SMN1*/*SMN2* copy number analysis was performed by the DRAGEN SMN Caller (version 4.0.3 and version 4.2.4)^10,26^, which requires at least 30x coverage. The SMN Caller calculates read counts aligning to *SMN1* and *SMN2*, normalises them using 3000 preselected genomic regions with stable coverage, and applies a Gaussian mixture model to infer copy numbers. *SMN1* copy number is determined using eight predefined differentiating sites, with copy number calls validated at a posterior probability threshold. Additionally, the SMN Caller detects truncated *SMN2* variants with partial exon 7 and 8 deletions by calculating total and intact *SMN* copy numbers. SMA carriers were defined as having one copy of the *SMN1* gene, and patients with SMA were identified as having zero copies. Copy number calls were further validated using MLPA (P021 kit, MRC Holland), including all detected SMA carriers and randomly selected non-carriers with varying *SMN1/SMN2* copy number combinations (Table S2).

Given the high sequence homology and suboptimal mapping quality of *SMN1* and *SMN2*, all detected variants were manually reviewed in the IGV before being recorded in the Variant Call Format. Pathogenicity predictions were conducted using TAIGenomics’ GenDiseak platform (v2.1.0) and VarSome (v11.15).

## Supplementary information

Supplementary Information

## Data Availability

The data supporting this study’s findings are available from the corresponding author upon reasonable request. Public availability of the data is contingent upon data release approval from the Taiwan Biobank and will be implemented at that time.
