# Supplementary material for: Application of whole genome sequencing for carrier and diagnostic assessment of spinal muscular atrophy in Taiwan
Source: NPJ Genom Med. 2025 Oct 29;10:69. doi: 10.1038/s41525-025-00524-1 (PMC12572182; doi:10.1038/s41525-025-00524-1)
Supplement: Supplementary file 1 — Supplementary Information [file 41525_2025_524_MOESM1_ESM.pdf]

Table S1. Carrier frequency of spinal muscular atrophy in East Asian populations

Reference	Ethnicity	Total samples	Method	Carrier frequency
2004 Chan (Hong Kong)[S1]	Southern Chinese	569	Real-time qPCR	1.58% (1 in 63)
2004 Lee (Korea)[S2]	Seoul	326	Real-time PCR	2.15% (1 in 47)
2005 Chen (China)[S3]	Fujian	264	Real-time fluorescent PCR	1.89% (1 in 53)
2007 Huang (Taiwan)[5]	Taiwan	310	MLPA	1.94% (1 in 52)
2010 Zhu (China)[S4]	Guangdong	1712	DHPLC	2.39% (1 in 42)
2010 Yoon (Korea)[S5]	Seoul	100	MLPA	2.00% (1 in 50)
2011 Chen (Taiwan)[S6]	Taiwan	163	Multiplex PCR and CE	2.45% (1 in 41)
2011 Su (Taiwan)[2]	Taiwan	107611	DHPLC and MLPA	2.10% (1 in 48)
2013 Gong (China)[S7]	Shanghai	4719	DHPLC and Multiplex PCR	1.91% (1 in 52)
2013 Qu (China)[S8]	Shanghai	1741	Real-time Fluorescent PCR	2.58% (1 in 39)
2014 Jing He (China)[S9]	Fujian	421	MLPA	3.09% (1 in 32)
2014 Zeng (China)[S10]	Sichuan	427	DHPLC	2.11% (1 in 47)
2015 Wang (Taiwan)[S11]	Taiwan	453	HRMA, DHPLC and MLPA	3.97% (1 in 25)
2018 Tan (China)[S12]	Guangxi	4931	DHPLC and Multiplex PCR	1.24% (1 in 81)
2020 Park (Korea)[S13]	South Korea	1581	MLPA	1.83% (1 in 55)
2020 Zhang J (China)[S14]	Nanjing	13069	qPCR and MLPA	1.77% (1 in 56)
2020 Zhang Y (China)[S15]	Yunnan	3049	MLPA	2.03% (1 in 49)
2021 Zhao (China)[S16]	Southern China	10585	NGS and Real-time PCR	1.40% (1 in 74)
2021 Chan (Hong Kong)[S17]	Hong Kong	143	NGS	2.1% (1 in 48)
2023 Huang (China)[S18]	Guangdong	5200	qPCR and NGS	1.44% (1 in 69)
2024 Sun (China)[S19]	Wuhan	18808	qPCR and MLPA	1.73% (1 in 58)
2024 Zhang L (China)[S20]	Guangdong	22913	qPCR and MLPA	1.70% (1 in 59)
Our study	Taiwan	1480	WGS and MLPA	1.55% (1 in 64)

Abbreviations: qPCR, quantitative real-time polymerase chain reaction; MLPA, Multiplex ligation-dependent probe amplification; DHPLC, denaturing high-performance liquid chromatography; CE, capillary electrophoresis; HRMA, high-resolution melting analysis; NGS, next-generation sequencing; WGS, whole-genome sequencing.

Table S2. Selected non-carrier samples with different copy number combinations for MLPA validation

Subject No.	1	2	3	4	5	6	7	8	9	10
***SMN1* CN**	2	2	2	3	3	3	3	4	4	4
***SMN2* CN**	1	1	2	0	1	2	3	0	1	2

Note: Ten non-carriers with various copy number combinations were selected for MLPA validation. The *SMN1* copy number exhibited a wider range of variation compared to the *SMN2* gene.
Abbreviations: SMN, survival motor neuron; CN, copy number; MLPA, multiplex ligation-dependent probe amplification.

Table S3. Distribution of *SMN1* and *SMN2* copy number types in the previous study

N (%)	***SMN1* CN0**	**CN1**	**CN2**	**CN3**	**CN4**	**Total**
***SMN2* CN0**	0	9 (0.01)	3631 (3.37)	1337 (1.24)	197 (0.18)	5174 (4.81)
**CN1**	0	313 (0.29)	30417 (28.27)	3614 (3.36)	0	34344 (31.91)
**CN2**	0	953 (0.89)	61035 (56.72)	3069 (2.85)	0	65057 (60.46)
**CN3**	1 (0.00)	981 (0.91)	2045 (1.90)	0	0	3027 (2.81)
**CN4**	3 (0.002)	6 (0.01)	0	0	0	9 (0.01)
**Total**	4 (0.004)	2262 (2.10)	97128 (90.26)	8020 (7.45)	197 (0.18)	107611

Modified from Su, Y. N. *et al.* Carrier screening for spinal muscular atrophy (SMA) in 107,611 pregnant women during the period 2005–2009: a prospective population-based cohort study. *PLoS One* **6**, e17067 (2011).
Abbreviations: SMN, survival motor neuron; CN, copy number.

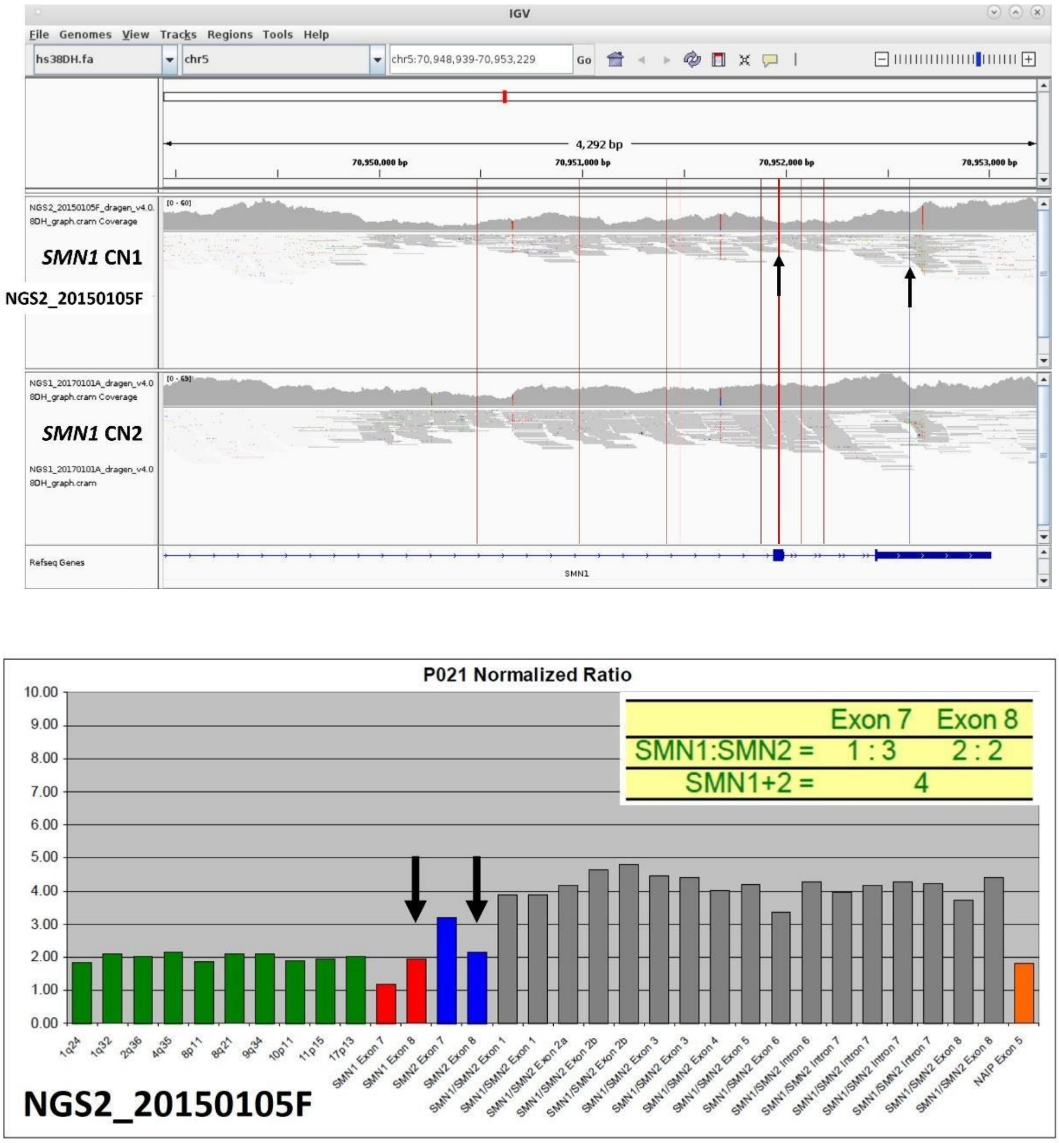

Figure S1-1 and S1-2. MLPA and IGV-based evaluation of a sample with suspected *SMN2* exon gene conversion to *SMN1*

(S1-1) MLPA profile revealed a discrepancy between exon 7 and exon 8, while exon 8 showed a higher copy number of the *SMN1* gene. The black arrows indicate the signals of exon 8 on *SMN1* and *SMN2* genes.

(S1-2) IGV review of WGS data from the same sample. Red lines mark eight differentiating sites used by the SMN Caller; the site within exon 8 is marked in blue. The black arrow highlights increased read depth at this position, consistent with a possible gene conversion event.

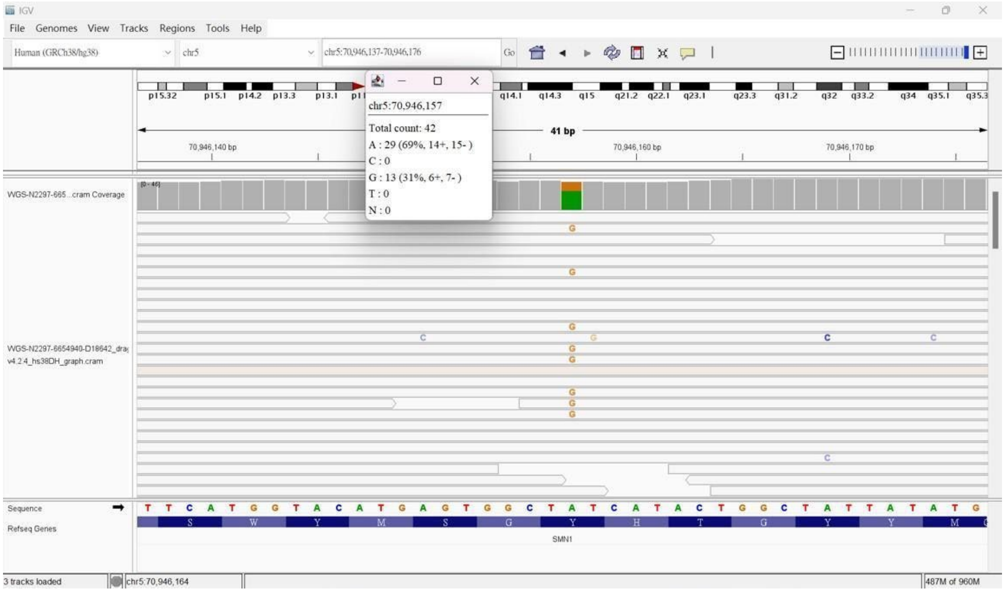

Figure S2. IGV plot of the missense variant, *SMN1*,c.815A>G

A single nucleotide substitution from adenine (A) to guanine (G) was identified at position 70,946,157 on chromosome 5, with a variant allele frequency of 31%. IGV, Integrative Genomics Viewer; SMN, survival motor neuron.

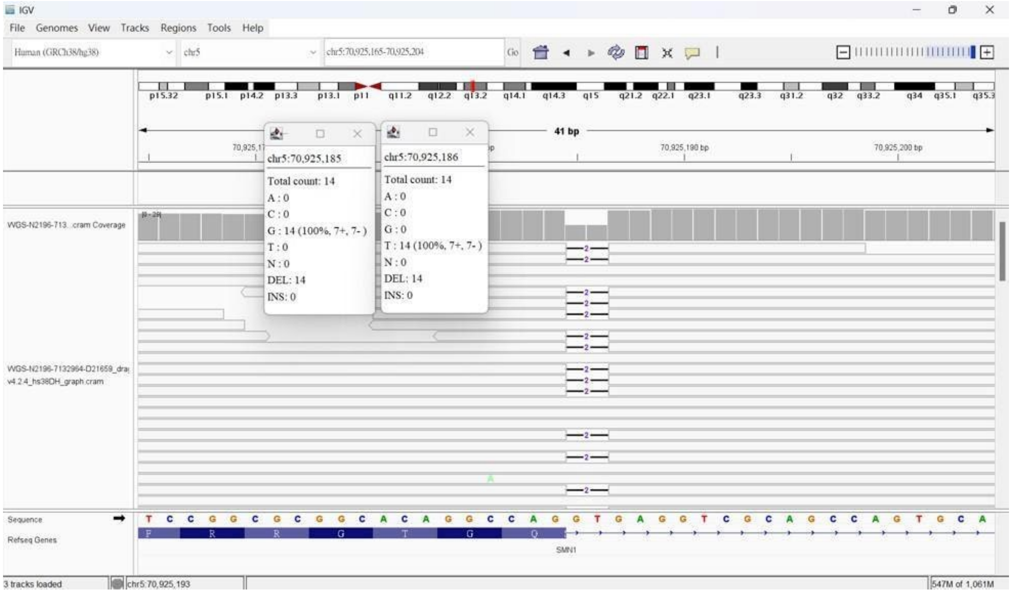

Figure S3. IGV plot of suspected splicing variant, *SMN1*, c.81+2_3delTG

A 2bp GT deletion was identified at positions 70,925,185 and 70,925,186 on chromosome 5, with a variant allele frequency of 50%. The deletion’s position and nucleotides on the IGV plot differed from the annotation due to the HGVS 3’ rule. IGV, Integrative Genomics Viewer; SMN, survival motor neuron; HGVS, Human Genome Variation Society.

References

S1. Chan, V., *et al.* Carrier incidence for spinal muscular atrophy in Southern Chinese. *J. Neurol.* **251**, 1089–1093 (2004).

S2. Lee, T. M., *et al.* Quantitative analysis of *SMN1* gene and estimation of *SMN1* deletion carrier frequency in Korean population based on real-time PCR. *J. Korean Med. Sci.* **19**, 870–873 (2004).

S3. Chen, W. J., Wu, Z. Y., Wang, N., Lin, M.-T. & Mu-rong, S.-X. Quantitative studies on *SMN1* gene and carrier testing of spinal muscular atrophy. *Zhonghua Yi Xue Yi Chuan Xue Za Zhi* **22**, 559–602 (2005).

S4. Sheng-Yuan, Z. *et al.* Molecular characterization of *SMN* copy number derived from carrier screening and from core families with SMA in a Chinese population. *Eur. J. Hum. Genet.* **18**, 978–984 (2010).

S5. Yoon, S., Lee, C. H. & Lee, K. A. Determination of *SMN1* and *SMN2* copy numbers in a Korean population using multiplex ligation-dependent probe amplification. *Korean J. Lab. Med.* **30**, 93–96 (2010).

S6. Chen, T. H., *et al.* Identification of bidirectional gene conversion between *SMN1* and *SMN2* by simultaneous analysis of *SMN* dosage and hybrid genes in a Chinese population. *J. Neurol. Sci.* **308**, 83–87 (2011).

S7. Gong, B., *et al.* Carrier screening for spinal muscular atrophy in 4719 pregnant women in shanghai region. *Zhonghua Yi Xue Yi Chuan Xue Za Zhi* **30**, 670–672 (2013).

S8. Qu, X. X. *et al.* A pilot study on spinal muscular atrophy carrier screening in shanghai region using real-time PCR. *Zhonghua Yi Xue Yi Chuan Xue Za Zhi* **30**, 1–4 (2013).

S9. He, J., Yao, X., Zhang, Q., Wang, N. & Chen, W. Screening of spinal muscular atrophy carriers in Fujian District by the multiple linkage dependent probe amplification technology. Paper presented at *The* 17th National Conference on Neurology of the Chinese Medical Doctor Association (Xiamen, Fujian, China, 2014)

S10. Zeng, G., *et al.* Analysis and carrier screening for copy numbers of *SMN* and *NAIP* genes in children with spinal muscular atrophy. *Zhonghua Yi Xue Yi Chuan Xue Za Zhi* **31**, 152–155 (2014).

S11. Wang, K. C. *et al.* Evaluation and characterization of a high-resolution melting analysis kit for rapid carrier-screening test of spinal muscular atrophy.

J. Neurogenet. **29**, 113–116 (2015).
S12. Tan, J., *et al.* Screening for spinal muscular atrophy mutation carriers among 4931 pregnant women from Liuzhou region of Guangxi. *Zhonghua Yi Xue Yi Chuan Xue Za Zhi* **35**, 467–470 (2018).
S13. Park, J. E. *et al.* Carrier frequency of spinal muscular atrophy in a large-scale Korean population. *Ann. Lab. Med.* **40**, 326–330 (2020).
S14. Zhang, J., *et al.* Carrier screening and prenatal diagnosis for spinal muscular atrophy in 13,069 Chinese pregnant women. *J. Mol. Diagn.* **22**, 817–822 (2020).
S15. Zhang, Y., *et al.* Result of carrier screening for spinal muscular atrophy among 3049 reproductive-age individuals from Yunnan region. *Zhonghua Yi Xue Yi Chuan Xue Za Zhi* **37**, 384–388 (2020).
S16. Zhao, S., *et al.* NGS-based spinal muscular atrophy carrier screening of 10,585 diverse couples in China: A pan-ethnic study. *Eur. J. Hum. Genet.* **29**, 194–204 (2021).
S17. Chan, O. Y. M., *et al.* Expanded carrier screening using next-generation sequencing of 123 Hong Kong Chinese families: A pilot study. *Hong Kong Med. J.* **27**, 177–183 (2021).
S18. Huang, Z., *et al.* Screening and prenatal diagnosis of survival motor neuron gene deletion in pregnant women in Zhaoqing City, Guangdong Province. *BMC Med. Genomics* **16**, 39 (2023).
S19. Sun, Y., *et al.* Preconception or prenatal acceptance of *SMN1* gene carrier screening and carrier rate of spinal muscular atrophy: A retrospective study in 18,818 reproductive age women in wuhan area of china. *J. Assist. Reprod. Genet.* **41**, 127–133 (2024).
S20. Zhang, L., et al. Carrier screening for spinal muscular atrophy in 22,913 Chinese reproductive age women. *Mol. Genet. Genomic Med.* **12**, e2359 (2024).
